# GridiLoc: A Backtracking Grid Filter for Fusing the Grid Model with PDR Using Smartphone Sensors

**DOI:** 10.3390/s16122137

**Published:** 2016-12-15

**Authors:** Jianga Shang, Xuke Hu, Wen Cheng, Hongchao Fan

**Affiliations:** 1 Faculty of Information Engineering, China University of Geosciences, Wuhan 430074, China; jgshang@cug.edu.cn (J.S.); chengwen@2014.cug.edu.cn (W.C.); 2 National Engineering Research Center for Geographic Information System, Wuhan 430074, China; 3 Institute of Geography, Heidelberg University, Berliner Street 48, Heidelberg D-69120, Germany; hongchao.fan@uni-heidelberg.de

**Keywords:** indoor localization, pedestrian dead reckoning, grid filter, backtracking, smartphone sensors

## Abstract

Although map filtering-aided Pedestrian Dead Reckoning (PDR) is capable of largely improving indoor localization accuracy, it becomes less efficient when coping with highly complex indoor spaces. For instance, indoor spaces with a few close corners or neighboring passages can lead to particles entering erroneous passages, which can further cause the failure of subsequent tracking. To address this problem, we propose GridiLoc, a reliable and accurate pedestrian indoor localization method through the fusion of smartphone sensors and a grid model. The key novelty of GridiLoc is the utilization of a backtracking grid filter for improving localization accuracy and for handling dead ending issues. In order to reduce the time consumption of backtracking, a topological graph is introduced for representing candidate backtracking points, which are the expected locations at the starting time of the dead ending. Furthermore, when the dead ending is caused by the erroneous step length model of PDR, our solution can automatically calibrate the model by using the historical tracking data. Our experimental results show that GridiLoc achieves a higher localization accuracy and reliability compared with the commonly-used map filtering approach. Meanwhile, it maintains an acceptable computational complexity.

## 1. Introduction

In ubiquitous and mobile computing, location is the most fundamental element [1]. Indoor environments are the main scenarios of people’s activity, and it is estimated that people spend about 87 percent of their time indoors [2]. While location-based context-aware systems, such as navigation and goods recommendation, are extending from outdoor spaces to indoor spaces [3], inferring the accurate locations of people in indoor spaces is still challenging. One of the commonly-used indoor localization methods is Pedestrian Dead Reckoning (PDR) [4], which has become a mainstream method due to the advent of sensor-rich smartphones. However, PDR often suffers from the accumulative error problem because of the drift in gyroscope readings and the influence of ferromagnetic materials on magnetometer readings, which may cause a large error in the heading estimation. A common solution to this issue is to combine map filtering with PDR [5,6,7]. Map filtering uses Bayesian techniques to reduce the uncertainty of indoor location estimations that violate the spatial constraints (e.g., walking through walls or obstacles), thereby enhancing the localization accuracy. However, map filtering takes into account a limited number of spatial contexts, and the definition of violating the spatial constraints is also imprecise. For instance, in the map filtering approach, wall-crossing estimations are identified if the line segment between two successive estimations intersects with a wall, which ignores the case of wall bypassing. Furthermore, it has low efficiency in performing frequent and time-consuming spatial computations by using a simple map, such as calculating the shortest path distance. The computational results can be used to aid indoor localization and are requested multiple times in one estimation. Furthermore, map filtering might encounter tracking failure issues: the accumulative error still exists in map filtering-aided PDR, especially in extremely complex indoor spaces (e.g., those spaces with many close turning corners or neighboring passages) where all of the particles may enter erroneous passages.

Indoor spatial models take into account more abundant spatial contexts than simple floor plans, which play a vital role in indoor localization, navigation and other location-based services [8,9,10]. Typically, spatial contexts can be divided into three categories: geometry, topology and semantics. In localization fields, two of the most important spatial models are graph models [11] and grid models [12]. The former uses nodes and edges to represent and discretize indoor spaces, while the latter tessellates indoor spaces into regular cells that contain semantic meanings (e.g., walls, obstacles, corridors) and have the exact same shape and size (typically between 10 cm and 1 m). Compared to the graph model, the most notable advantage of the grid model is its capability of representing accurate locations since it has a small cell size, which contributes to the reachability of almost any locations of an indoor environment. Moreover, the grid model is well-suited to efficient computations, as it can be seen as a matrix that enables matrix-based computations [12]. In addition, the grid model can be readily constructed based on a floor plan by using existing off-the-shelf Geoinformatic Information System (GIS) software, such as ESRI (Redlands, CA, USA) ArcGIS [13]. Therefore, the grid model has been widely used in robotics tracking, artificial intelligence and fingerprinting.

In this paper, we propose GridiLoc, a reliable and accurate pedestrian indoor localization method via combining the grid model and PDR. This work is motivated by the occupancy grid filter, which was initially proposed in the communities of mobile robotics and artificial intelligence [14]. GridiLoc uses the grid model to represent fine-grained indoor spatial contexts, including geometries (e.g., the shortest path distance), topologies (e.g., connectivity and proximity) and semantics (e.g., walls, obstacles, stairs and elevators), which are beneficial to the improvement of PDR.

As a preview, our main contributions are as follows:

1. We innovatively combine the grid filter and PDR for indoor localization on smartphones. The proposed algorithm can achieve accurate pedestrian tracking by using the grid model and the inertial sensors embedded in smartphones, without relying on any wireless infrastructures.
2. We propose a backtracking grid filter algorithm. The historical tracking data are used to tackle the dead ending issues, thereby eliminating the tracking failure caused by the accumulative error of PDR. Meanwhile, a topological graph is introduced for representing candidate backtracking points, which can largely reduce the time consumption of backtracking. In certain cases, the historical tracking data can also be used to automatically calibrate the step length model of PDR.
3. We evaluate the accuracy, reliability and computational complexity of GridiLoc in a multi-floor experimental environment. Experimental results show the GridiLoc achieves a higher accuracy and reliability than the map filtering approach, especially in complex indoor environments. We implement GridiLoc, which runs smoothly on Android smartphones, proving its low and acceptable computational complexity.

## 2. Related Works

Map-aided localization: Map-aided localization can be categorized into two types: map matching [15,16] and map filtering [5,6,7]. Lan et al. [15] proposed a map matching method, which matches estimated trajectories to the true passages in maps to find the last visited corners. Subsequently, the heading estimation error caused by gyroscopes can be calibrated. Compared to map matching, map filtering is more common and effective, which uses Bayesian filters to rule out the locations where users are impossible to be (e.g., walls and obstacles). Map filtering can considerably improve the indoor localization accuracy, without demanding extra investments in infrastructures. It therefore has become a popular technique for improving the location estimation amongst prior research. However, map filtering takes into account a limited number of spatial contexts, and the definition of the spatial constraint violation is imprecise. For example, users can enter a room through a door so as to bypass a wall. In this case, it is problematic to simply identify wall-crossing estimations by checking whether the line segment between two successive estimations intersects with a wall. Furthermore, it has low efficiency in performing frequent and time-consuming spatial computations by using a simple map, such as to calculate the shortest path distance between two locations. The computational results can be utilized to aid PDR and are requested multiple times in one estimation. Besides, tracking failure occurs frequently in PDR, but map filtering is unable to tackle this issue well. For instance, when a user is taking a turn around a corner, a small or large step length model parameter might cause all of the particles to hit walls. Furthermore, an incorrect heading estimation will cause particles to propagate into erroneous passages if multiple passages exist nearby, ultimately leading to the death of all of the particles. When this issue occurs, map filtering fails to calibrate the estimation towards an expected location.

Grid model-aided localization: Typically, the grid model is used in robotics tracking. Fox et al. [14] proposed a grid-based Markov localization algorithm, which uses a fine-grained grid model to represent a robot’s state space. When the robot receives new sensor measurements (e.g., ultrasound sensor readings), the probability of each state that is represented by a grid cell will be updated. To update the probability more efficiently, two strategies are adopted: pre-computation of sensor models and selective update. In indoor localization fields, grids can also act as the basic units that represent indoor locations. For instance, occupation grids are often used in Wi-Fi fingerprinting to represent the locations of fingerprints. Bhattacharya et al. [17] proposed using a grid model to represent the indoor space of a grocery store, which is divided into grid cells. The occupation grid can be used to limit a target’s estimation, thereby improving the localization accuracy of Wi-Fi fingerprinting. The researchers in [18,19,20] proposed using Bayesian techniques to fuse multiple sensors and/or opportunity wireless signals, such as Inertial Measurement Units (IMUs), Wi-Fi and cameras, and utilizing a 2D grid model with a fixed cell size to represent indoor spaces.

To sum up, grid-aided localization algorithms are mostly used in robotics. However, considerable differences exist between robotics and pedestrians with regard to localization. For instance, the former normally covers a limited area of a single floor, while the latter needs the support of multiple-floor tracking. Furthermore, the former requires measurements from laser or sonic rangefinders, while the latter simply relies on the sensors of hand-held mobile devices. Therefore, the localization methods in robotics are unable to be applied directly to pedestrian tracking. Existing research on pedestrian tracking mainly focuses on the utilization of the grid model to improve wireless localization (e.g., Wi-Fi fingerprinting) rather than PDR. It is meaningful, but challenging to investigate how a grid model can be used to improve the location estimation of PDR on smartphones without the assistance of external infrastructures and other localization hardware.

## 3. Overview of GridiLoc

**Figure 1:**
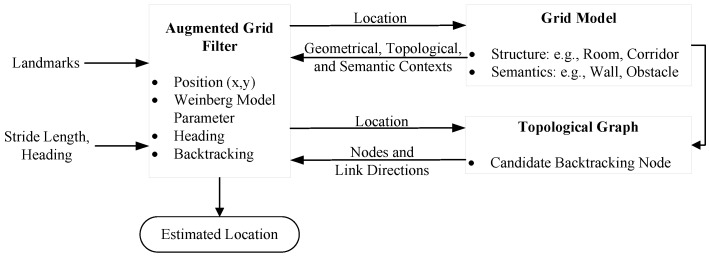
Architecture of GridiLoc.

The architecture of GridiLoc is illustrated in Figure 1. The key idea of GridiLoc is the fusion of PDR and a grid model. The grid model can limit users’ movement in indoor environments, thereby improving the location estimation of PDR.

GridiLoc consists of five key steps: initialization, prediction, update, landmark detection and backtracking. In the initialization step, the probability grid $\left\{p_{k,0}\right\}$ is initialized, which is then used as the input in the prediction step to predict users’ initial location ($x_{t}=1$). In the prediction step, the prior probability is calculated based on the previous location, the grid model and the estimated stride length and heading. The step length and the heading are sampled in the Gaussian distribution, and several candidate cells may be obtained. In the update step, the posterior probabilities of these candidate cells are updated by using the grid model, which is followed by the normalization of the probability. The current estimation is thus the weighted mean of these candidate cells. The posterior probability of a candidate cell is determined by two factors. One is the property of the candidate cell (e.g., walls or obstacles). The other is the difference between the shortest path distance and the Euclidian distance between the candidate cell and the previous state. If the posterior probabilities of all of the candidate cells are zero, then a dead ending event occurs.

If the dead ending state lasts for some time, backtracking will be invoked. Backtracking uses the historical tracking data and a topological graph to find the backtracking point, which is used to calibrate the estimation at the starting time of the dead ending. If the dead ending is caused by an erroneous step length model, the original model parameter (Weinberg model [21]) will be automatically calibrated by using the historical tracking data. More specifically, given the location of the last visited topology node and the backtracking point, we can obtain the expected distance a user has traveled during this period of time. The estimated distance is calculated by using the original step length model parameter. In this way, the expected step length model parameter equals the result of dividing the product of the expected distance and the original step length model parameter by the estimated distance.

When a user is detected moving in vertical passages, such as stairs and elevators, his or her location estimation will be calibrated. Current floor level will be calculated when the user leaves the vertical passages. The detailed description of the landmark identification and the floor level detection can be found in [22].

The pseudocode below describes the GridiLoc algorithm (Algorithm 1). It consists of four key steps: initialization, prediction, update and backtracking, the details of which are discussed in the next sections. In the pseudocode, the ChangedLevel function corresponds to the floor level capture algorithm in [22].

**Algorithm 1.** GridiLoc ($x_{0}$, $l_{0}$, M, G, z, r).**INPUT:** A user’s initial location $x_{0}$ on the $l_{0}$-th floor, the grid model M, the topological graph G, a set of estimated step lengths and headings z and the raw sensor data r from Time 1 to N.**OUTPUT:** The trajectory $tr=\{x_{1},x_{2},\ldots x_{N}\}$.$[\{p_{k,0}\},M_{l_{0}}]\leftarrow$ Initialization ($x_{0}$, $l_{0}$, M); {Initialize the probability grids}$v\leftarrow x_{0}$; {The last visited topology node}**for**
*t*
← 1 to N **do**  **if** the movement on stairs or elevators between time m and n is captured, **then**    $x_{t}$ is set as the location of the captured landmark;    $v\leftarrow x_{t}$;    $l_{0}\leftarrow$$l_{0}$ + ChangedLevel ($\left\{r_{i},m<i<n\right\}$);     $[\{p_{k,0}\},M_{l_{0}}]\leftarrow$ Initialization($x_{t}$, $l_{0}$, M);     **continue**;  **end**;    **if** a turning corner event is captured, **then**    v is set as the location of the captured landmark;  **end**;  $\{p_{k,t}\}\leftarrow$ Prediction ($\left\{p_{k,t-1}\right\}$, $x_{t-1}$, $z_{t}$);   $[x_{t},\{p_{k,t}\}]\leftarrow$ Update ($\left\{p_{k,t}\right\}$, $M_{l_{0}}$);   **if** T continuous dead ending events are captured, **then**    Let S and O be the step lengths and headings from time (*t* – *T* + 1) to *t*;    $x_{t-T+1}\leftarrow$ Backtracking (G, v, $x_{t-T+1}$, S, O);    Recalculate the user’s trajectory from time *t* – *T* + 2 to *t*;   **end;****end; {**of **for}**

## 4. PDR-Based Grid Filter

The grid model divides an indoor space into regular grid cells with an equal size (e.g., 0.7 m). The indoor space is not abstracted in the grid model, which allows one to accurately and continuously represent the locations of most objects. Each cell contains the probability that the person or object is in it. The probability of the cells containing static objects (e.g., furniture, walls) is set to zero since they are unreachable.

### 4.1. Initialization

This stage initializes the probabilities of all cells. The probability of a cell will be set to one if a user’s initial location lies in this grid cell, otherwise, zero. We then record the index or ID of the cell with a probability of one, and this index is used in the prediction stage to generate a buffer.

### 4.2. Prediction

**Figure 2:**
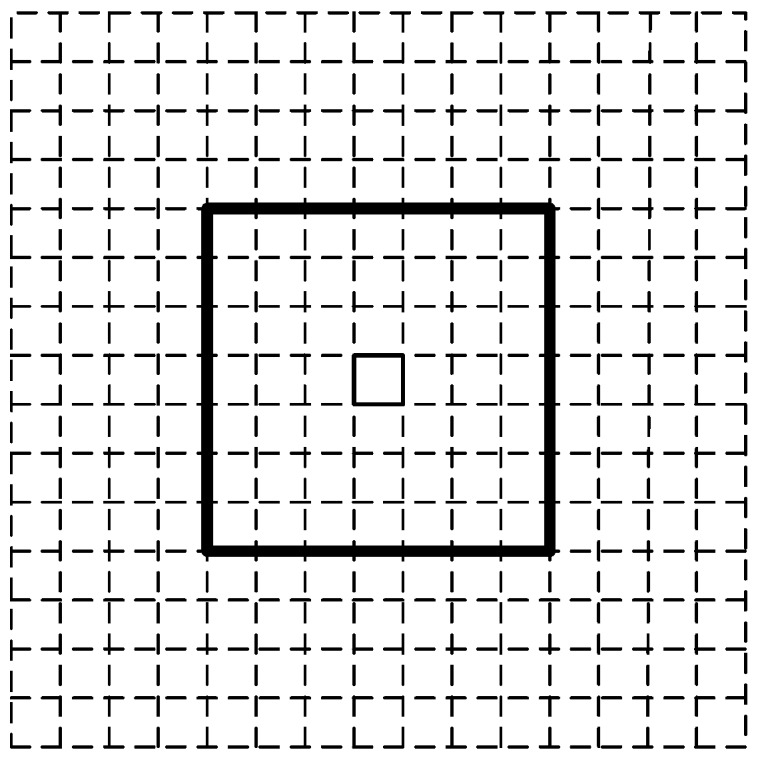
An example of a buffer.

According to the user’s previous state and the currently estimated step length and heading, the candidate cells of the current state can be obtained. Then, the prior probabilities of these candidate cells are calculated by using the formula ${p^{-}}_{k,t}=p(x_{t}|x_{t-1},z_{t})$. Specifically, this stage includes two steps:

- Determine a buffer: Normally, the grid filter needs to traverse all of the cells of the grid model in order to calculate the probability of each cell. However, the computational load becomes quite high when the number of cells is huge. To reduce the computational load, a buffer mechanism is introduced. A buffer is defined as a subset of a complete grid model, and it represents the search region of a user’s current state. The range of a buffer depends on the user’s previous location and walking speed. As depicted in Figure 2, the buffer in this paper is denoted by a rectangle with a side length of seven-times the cell size (0.7 m) and centering at the previous state, given the fact that a user’s walking distance in one step cannot be over two meters. In the prediction and update stage, we just need to calculate the probabilities of the cells within the buffer and rule out the other cells directly.
- Calculate the prior probability: The estimated step length and heading derived from smartphone inertial sensors are generally inaccurate. Thus, they need to be sampled, and more than one candidate cell may be produced in the buffer. A pair of step length and heading can be represented by a directed line segment with the starting point as the center of the buffer, while the sampled ending point is determined by the sampled step length and heading. For the candidate cells in the buffer, their prior probabilities are calculated by using Equation (1), which equal the sum of the probabilities of the sampled ending points located in the candidate cells. The probability of a sampled ending point is the joint probability of its sampled heading and step length, denoted by $\bar{p}(d^{k})$ and $\bar{p}(\theta^{k})$, respectively. The Probability Density Function (PDF) of the step length and heading is approximated as a Gaussian distribution; thus, the probability of a sampled step length or heading can be calculated through Equation (2), where ${r_{t}}^{i}$ is the step length or heading of the *i*-th sample at time *t*. The standard deviation of the Gaussian distribution for the step length or heading is denoted by σ. The mean value of the step length or heading at time *t* is denoted by $z_{t}$, which is the estimated step length or heading at time *t*.

(1) $$p(g)=\sum_{k=1}^{n}\bar{p}(d^{k}).\bar{p}(\theta^{k})$$

(2) $$\bar{p}=\frac{1}{\sqrt{2\pi }\sigma }\exp\left[-\frac{{\left\|z_{t}-{r_{t}}^{i}\right\|}^{2}}{2\sigma^{2}}\right]$$

### 4.3. Update

**Figure 3:**
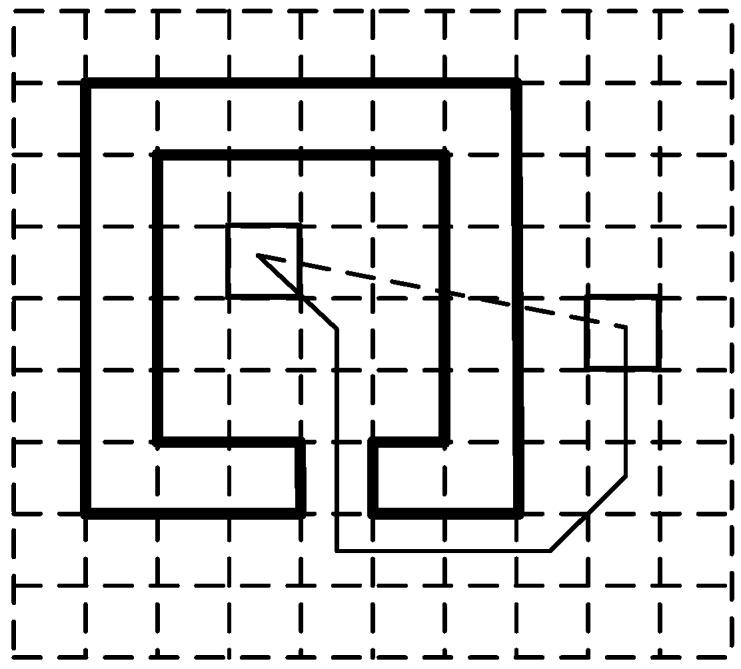
Euclidean distance and the shortest path distance between two cells.

This stage calculates the posterior probabilities of candidate cells, which is followed by the normalization of the posterior probabilities. The weighted mean of the centers of candidate cells is calculated, and the cell that contains the weighted mean is treated as the current estimation. Then, the probability of the cell representing the current estimation is set to one, and the probabilities of the other cells are set to zero. The posterior probability of a candidate cell is determined by two factors. One is the semantics of the cell. That is, if the property of a candidate cell is obstacles or walls, the probability of this cell is set to zero. The other is the difference between the shortest path distance and Euclidean distance between the candidate cell and the cell of the previous estimation. A large distance difference indicates the existence of many obstacles or a big obstacle between two cells, which means overpassing the obstacles in one step is almost impossible. Thus, the larger the distance difference, the lower the probability of a user moving from the cell of the previous estimation to the candidate cell. If the distance difference is over a certain threshold, the probability of the candidate cell will be set to zero. The shortest path distance is calculated by using the A* search algorithm [23]. Figure 3 shows an example of the shortest path distance denoted by a solid line and of the Euclidean distance denoted by a dotted line.

## 5. Backtracking

### 5.1. Dead Ending

**Figure 4:**
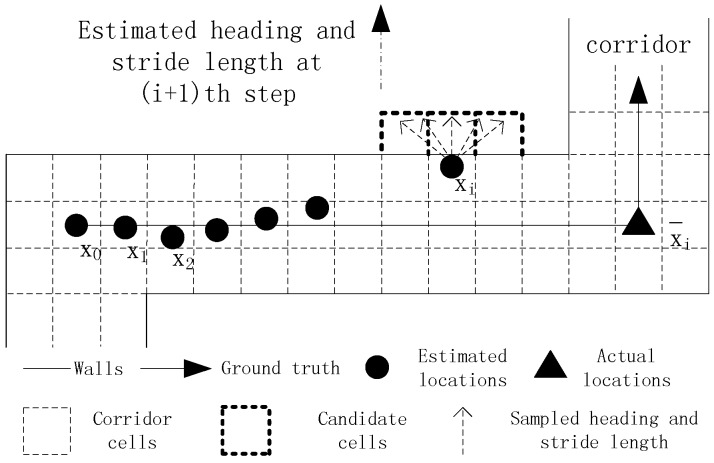
An example of dead ending.

The PDR method encounters the dead ending problem when testing environments are complex. If this issue is not tackled well, it may lead to the failure of successive tracking, as shown in Figure 4. The black solid line represents the ground truth, and the black solid dots represent the estimated locations. The initial location, true location at time *i* and estimated location at time *i* are denoted by $x_{0}$, ${\bar{x}}_{i}$ and $x_{i}$ respectively. Due to the use of an inaccurate step length model whose parameter is smaller than its real one, the estimated location at time *i* deviates from the true location. The target takes a turn around a corner at time *i* + 1, and the step length and heading are calculated, which are denoted by a line segment with a black solid arrow. Then, the grid filter is used to infer the target’s location at time *i* + 1. In the prediction stage of the grid filter, the estimated heading and step length are sampled according to a Gaussian distribution. Consequently, all of the sampled candidate cells fall into the other side of a wall. In the following update stage, the posterior probabilities of all of the candidate cells are set to zero because the shortest path distances between the cell of the previous estimation $x_{i}$ and all of the candidate cells are much larger than their Euclidean distances. Finally, the estimated location at time *i* + 1 remains the same as the estimated location at time *i*. This situation is called the dead ending in which a user is walking forward, but his or her current estimation remains unchanged.

Typically, the dead ending occurs at corners or forked passages, and tracking can leave the dead ending state only if the movement orientation changes. The dead ending issue is mainly caused by the accumulative error of PDR, which is caused by the erroneous step length model and heading estimation. For example, the dead ending event in Figure 4 is caused by an inaccurate step length model parameter. When the first dead ending event occurs, it will not be immediately processed, but be delayed until T successive dead ending events are captured. In our opinion, if tracking can leave the dead ending state in a short time, subsequent tracking will not be affected. Thus, it is needless to start the time-consuming backtracking process for dealing with short-time dead ending events.

### 5.2. Backtracking Algorithm

In topological graphs, indoor spaces are represented by nodes and links. Nodes depict key locations in indoor environments, such as rooms, elevators and stairs. Links represent connectivity among nodes, such as corridors and passages. To reduce the time consumed by the backtracking process, a topological graph is used to represent candidate backtracking points and the connectivity among them. The candidate backtracking point corresponds to a corner in an indoor environment, and the connectivity refers to a passage or a corridor that links candidate backtracking points.

When a user visits a topology node, the node will be recorded. If T successive dead ending events occur, backtracking will be started to handle this issue. The last visited node and the historical tracking data in the T dead ending states are fused to infer a correct backtracking point, which is actually the expected location at the starting time of the dead ending events. Starting from the backtracking point, the user’s trajectory in the dead ending states is recalculated. The detailed description of backtracking is as follows.

- First, the last visited topology node *v* is recorded and updated during tracking. When T successive dead ending events at location x have been captured, the heading estimation *h* on the path from *v* to x is then calculated. Based on node *v*, another node if found in the topological graph such that the node is linked to *v* and that the difference between the link direction and *h* is below a threshold H. This node is selected as an initial candidate node. If only one candidate node exists, this node will be treated as the backtracking point.
- Otherwise, the topology nodes linked to the candidate nodes are used to identify the correct candidate node, which are called association nodes. The average heading estimation in dead ending states is denoted by o, and the direction from a candidate node to its corresponding association node is denoted by $o^{\prime }$. The candidate node whose direction $o^{\prime }$ is closest to o is then treated as an optimal backtracking point.
- However, if there still exists more than one optimal backtracking points, the one with the nearest distance to x is chosen as the backtracking point.

According to the number of initial candidate nodes, backtracking can be categorized into two types: Single node and multiple nodes.

#### 5.2.1. Single Node

**Figure 5:**
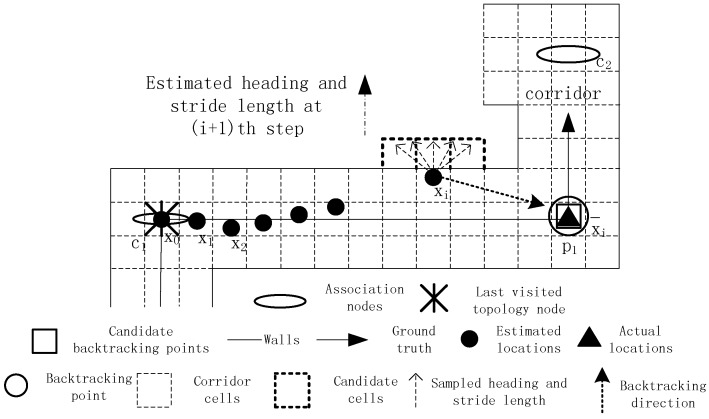
Backtracking with only one initial candidate node.

In this case, only one corner exists nearby, and the dead ending is very likely caused by an erroneous step length model parameter. The model parameter can be then calibrated by using the tracking data in dead ending states. In order to better describe the absolute directions used in our method, we assume there exists a virtual coordinate system in the test scene. In the coordinate system, the X-axis is set to the horizontal direction with its positive axis pointing from left to right. The Y-axis is set to the vertical direction with its positive direction upward. As shown in Figure 5, $x_{i}$ is the location where the dead ending occurs, and the estimations from time *i* to time *i* + *T* remain unchanged. Back to time *i*, the last visited topology node is $x_{0}$, and the direction from $x_{0}$ to $x_{i}$ is around zero degrees. According to the backtracking point selection algorithm, $p_{1}$ is regarded as the candidate node, as well as the final backtracking point. The location estimation at time *i* is then calibrated to $p_{1}$. Then, the historical tracking data from time *i* to time *i* + *T* are used to recalculate the target’s locations during this period.

The step length model parameter is updated according to the following equation:

(3) $$m^{\prime }=(s+d)\cdot \tilde{m}/s$$

where $\tilde{m}$ is the original step length model parameter, and *s* denotes the sum of the step length estimations from the time visiting the last corner (Time 0) to the time the dead ending starts (Time *i*). *d* denotes the distance between the true location at time *i* $p_{1}$ and $x_{i}$ where the dead ending occurs. *s* + *d* depicts the expected distance the target has traveled during the period from Time 0 to time *i*. Since the direction the target moves during this period is approximate to the direction from $x_{i}$ to $p_{1}$, the expected distance is the addition of *s* and *d*. If the two directions are opposite, the expected distance is *s* − *d*.

#### 5.2.2. Multiple Nodes

**Figure 6:**
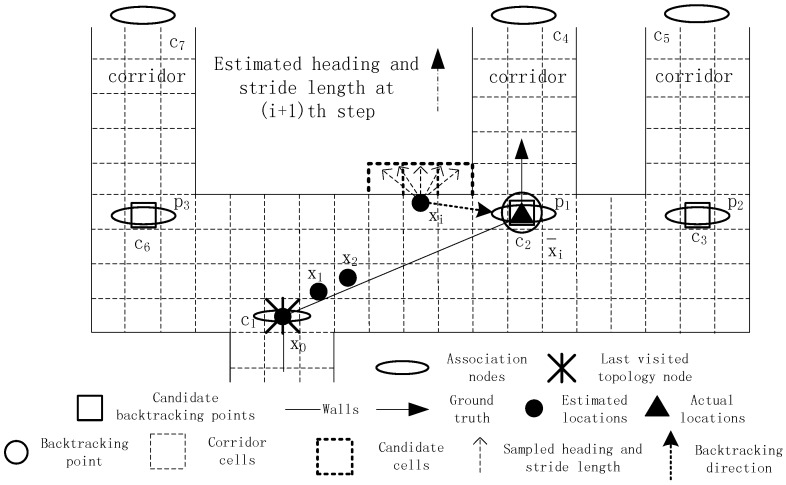
Backtracking with more than one initial candidate node.

In this case, several neighboring passages probably exist, and the heading error might be the main reason for the dead ending rather than the step length model. Therefore, the model parameter cannot be calibrated. As shown in Figure 6, the last visited topology node is $x_{0}$, and the location where the dead ending occurs is denoted by $x_{i}$. The direction from $x_{0}$ to $x_{i}$ is about 90°, while the directions from $x_{0}$ to $p_{1}$, $p_{2}$ and $p_{3}$ are all around 90°. Thus, $p_{1}$, $p_{2}$ and $p_{3}$ will be chosen as the initial candidate nodes. To identify the correct one from these candidate nodes, the corresponding association nodes including $c_{1}$, $c_{2}$, $c_{3}$, $c_{4}$, $c_{5}$, $c_{6}$ and $c_{7}$ are applied. Unfortunately, these three candidate nodes still cannot be distinguished because the directions from $p_{1}$ to $c_{4}$, $p_{2}$ to $c_{5}$ and $p_{3}$ to $c_{7}$ equal the walking direction in the dead ending states. However, because $p_{1}$ has a smaller distance to $x_{i}$ than $p_{2}$ and $p_{3}$, it is selected as the best backtracking point.

The pseudocode below describes the backtracking algorithm (Algorithm 2), where the UpdateStepModel procedure is the step length model update algorithm.

**Algorithm 2.** Backtracking (G, v, x, S, O).**INPUT:** A topological graph G, the last visited topology node v, the location where the dead ending occurs x, a set of estimated step lengths S and headings O during the dead ending state.**OUTPUT:** The inferred location $\bar{x}$ at the starting time of the dead ending.  B←null; {Candidate backtracking points}  Let h be the orientation from v to x;  **for**
∀(p,v)∈G, **do**    Let $h^{\prime }$ be the orientation from v to p;    **if**
$\left|h^{\prime }-h\right|<H$, **then** {H is a heading threshold}      B←B∪{p};    **end**;  **end**; {of **for**}  **if**
*size*(B) == 1, **then {** Only one element exists in B**}**    $\bar{x}\leftarrow B_{1}$;     UpdateStepModel (v, x, S, O);    **return**;  **end**  **else {**Association topology nodes are used as the criteria**}**   L←size(O);   $o\leftarrow \frac{\sum_{l=1}^{L}O_{l}}{L}$; {o is the mean value of the heading array O}   $B^{\prime }\leftarrow \{B_{1}\}$;   $d^{\prime }\leftarrow \max\_value$;   **for**
i← 1 to *size*(B) **do**     $p^{\prime }\leftarrow B_{i}$;     **for**
$\forall (p^{\prime },c)\in G$
**do**       Let $o^{\prime }$ be the orientation from $p^{\prime }$ to c;        $d=\left\|o^{\prime }-o\right\|$;        **if**
$d==d^{\prime }$
**then**          **if**
$p^{\prime }\notin B^{\prime }$
**then**            $B^{\prime }\leftarrow B^{\prime }\cup \{p^{\prime }\}$;           **end;**        **end;**        **if**
$d<d^{\prime }$
**then**          $d^{\prime }\leftarrow d$;         **if**
$p^{\prime }\notin B^{\prime }$
**then**           $B^{\prime }\leftarrow \{p^{\prime }\}$;          **end;**       **end;**     **end; {**of **for}**   **end; {**of **for}**  **end;** **if**
*size*($B^{\prime }$) == 1, **then** { Only one element exists in $B^{\prime }$}    $\bar{x}\leftarrow {B^{\prime }}_{1}$;     **return**; **end** **else** {The point $p^{\prime }$ in $B^{\prime }$ with the shortest path distance to x is picked}    $d^{\prime }=A^{*}({B^{\prime }}_{1},x)$; {Calculate the path distance with $A^{*}$ algorithm}    m=1;    **for** k ← 2 to *size*($B^{\prime }$), **do**      $d\leftarrow A^{*}(B_{k}^{\prime },x)$;      **if**
$d<d^{\prime }$, **then**        $d^{\prime }\leftarrow d$; m←k;      **end;**    **end**; {of **for**}    $\bar{x}\leftarrow {B^{\prime }}_{m}$;    **return**; **end;**

## 6. Experiments

A series of experiments was conducted in a real environment to evaluate the proposed GridiLoc method from five aspects: accuracy and reliability, the computational complexity, the impact of threshold *T*, the adaptivity of the step length model and the energy consumption. GridiLoc is compared with the approaches fusing the particle filter and maps (map filtering) [5,22]. For the map filtering approach, the numbers of particles of 50 and 1000 were tested. GridiLoc and map filtering used the same test data, the same PDR parameters, as well as the same landmark detection and calibration methods. The difference is that the grid filter uses grid models, while the map filtering approach uses floor plans.

### 6.1. Experimental Setup

#### 6.1.1. Experimental Testbed

**Figure 7:**
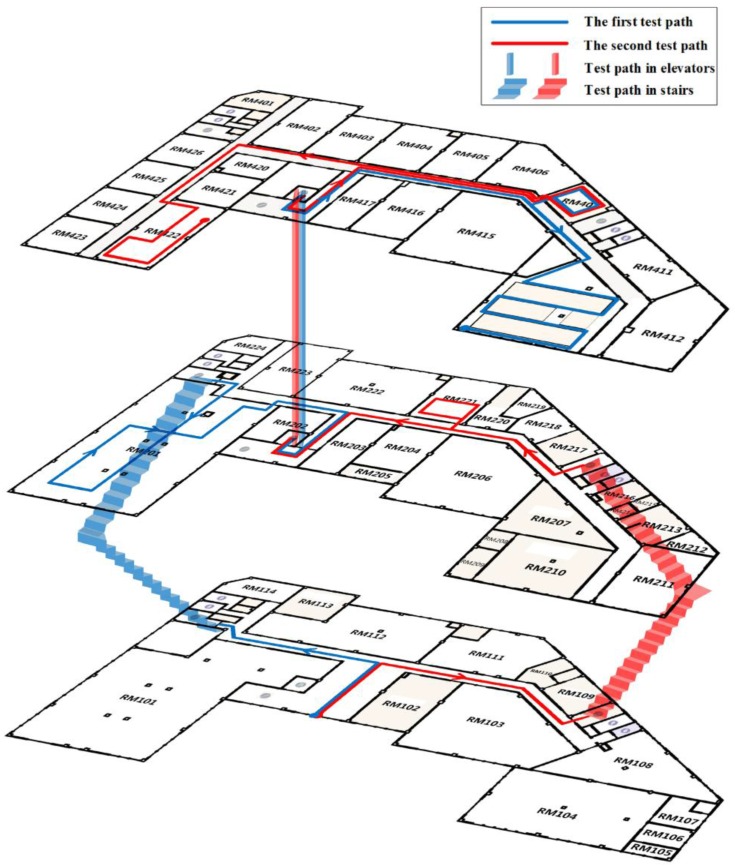
Testbed.

An office building occupied by the China National Engineering Research Center for Geographic Information System (GIS) is selected as our testbed. This building consists of four floors with 3300 square meters for each floor, including an elevator and three staircases. Figure 7 shows the experimental area where two test paths are marked in blue and red solid lines, respectively. The arrow on the paths represents the testers’ walking direction. The two paths cover three floors, starting from the first floor to the fourth floor. The first path in blue is 365 m long, while the other path in red is 345 m long. Three testers walked along the two paths carrying two different smartphones and collected 24 trajectories in total.

**Figure 8:**
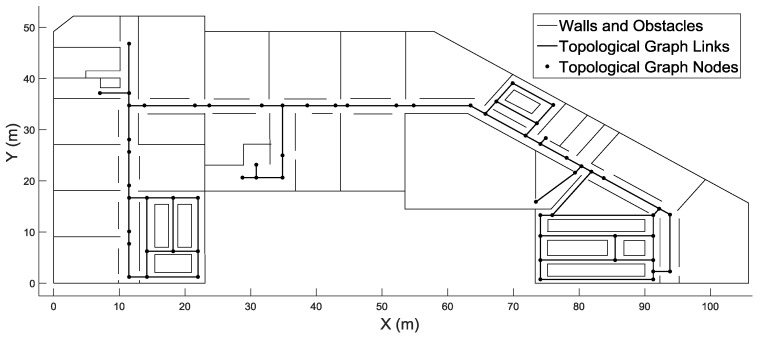
Topological graph on the fourth floor.

The inputs of our system are sensor data and spatial data, including a grid model, the locations of landmarks (stairs and elevators) and the topological graphs representing corners (nodes) and the connectivity (links) among them. The grid model was first generated by ArcGIS from floor plans. Based on the grid model, the locations of stairs, elevators and corners, as well as the connectivity among the corners were extracted. Figure 8 shows the topological graph on the fourth floor.

**Figure 9:**
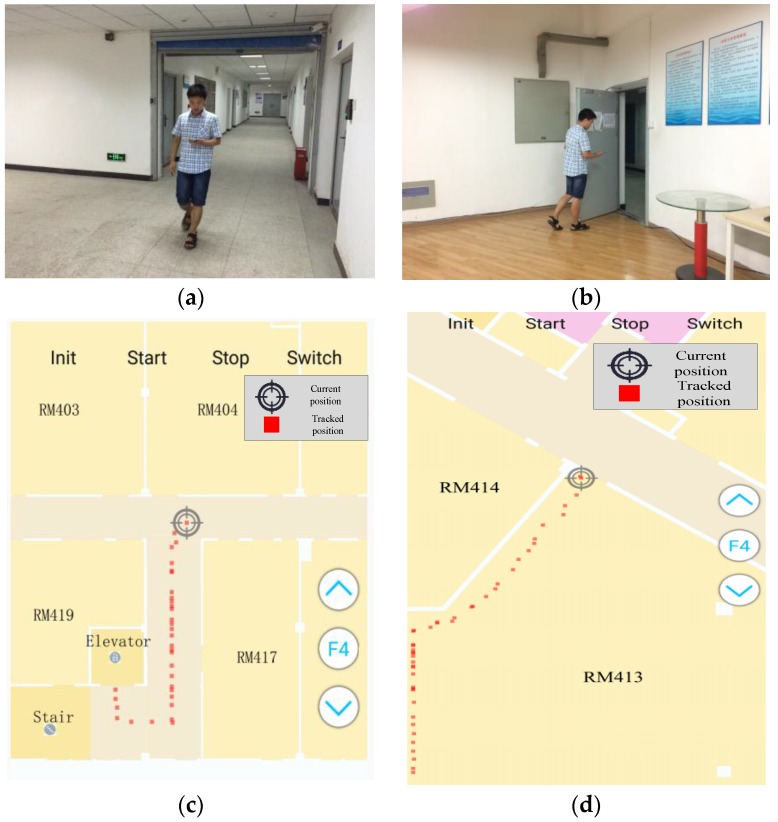
Snapshots of the data collection process and the corresponding screenshots of the in-time tracked trajectories on smartphones. (**a**) Walking down a corridor; (**b**) walking through a door; (**c**) tracking in the corridor; (**d**) tracking in the large room.

A prototype system has been built and tested on the Samsung Galaxy S Note 4 smartphone with a 2.7-GHz CPU and the Google Nexus 6 smartphone with a 2.7-GHz CPU. Figure 9a,b shows the snapshots of two test areas where one of the testers was walking down a corridor and walking through a door, respectively. Figure 9c,d shows the in-time trajectories in the two test scenes. The floor plans of the three floors were divided into small tiles (256 × 256) and stored on smartphones, while the grid models and the topological graphs were stored in the smartphone’s SQLite database. When the app was launched, a user could choose the map of their current scene and location by pinning the map. The app then started to track the user and update the location on a per step basis. Apart from tracking users online, raw sensor (e.g., accelerometers, gyroscopes, compasses and barometers) data were also automatically collected and stored on phones for the convenience of analyzing and evaluating the performance of different localization algorithms by offline processing.

#### 6.1.2. Parameter Setting

In the experiment, several key parameters need to be set properly. For PDR, inferring the step length and the heading is crucial. The step length was estimated with the Weinberg model, and the model parameter was set to 0.6. The heading was estimated with the Kalman filter by fusing the data from gyroscopes and compasses, and the process noise covariance and the measurement noise covariance of the Kalman filter were set to 0.00085 and 9.0657, respectively. For the grid filter, the estimated step length and heading were sampled according to a Gaussian distribution in the prediction stage, and the sample number was set to 100. The standard deviations of the estimated step length and heading were set to 0.2 (m) and 15°, respectively. The threshold *T* used to judge whether backtracking should be started was set to five.

### 6.2. Localization Accuracy and Reliability

Four error measurement methods are used to evaluate the localization accuracy of GridiLoc and map filtering: standard error, mean error, median error and 67% Circular Error Probable (CEP), as shown in Table 1.

**Figure 10:**
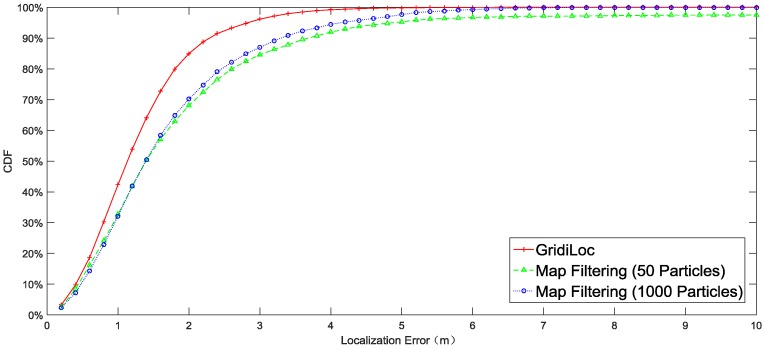
Performance comparison of different methods.

**Figure 11:**
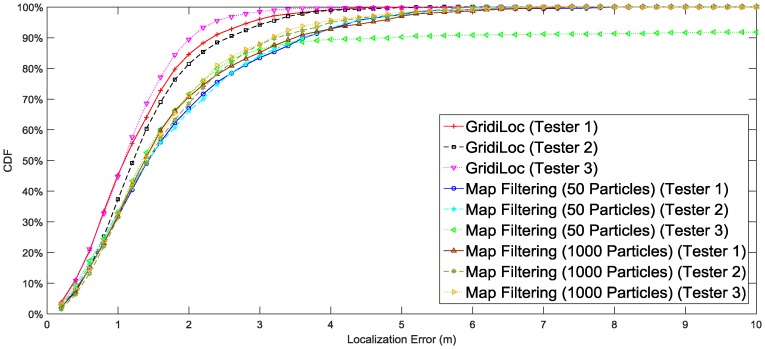
Performance comparison of different testers.

**Figure 12:**
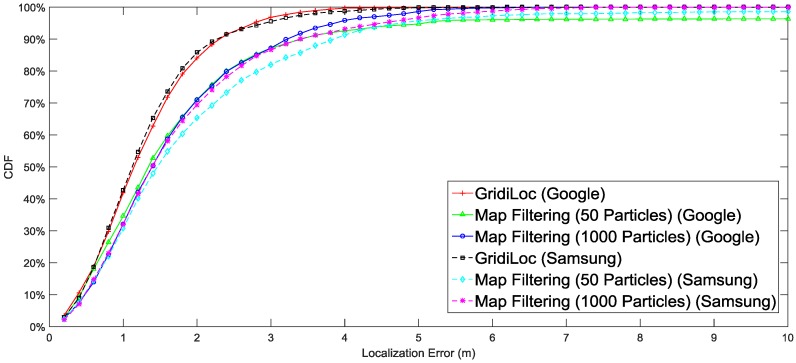
Performance comparison of different smartphones.

Figure 10 illustrates the localization result of different solutions. The result shows that our solution tracks users with an accuracy of 2.5 m 95% of the time, while map filtering with 1000 particles achieves only an accuracy of 4 m 95% of the time. When the particle number decreases to 50, map filtering achieves an accuracy of 5 m 95% of the time. In order to show the influence of different smartphones and different testers on the localization performance, the complete localization results in Figure 10 are separated according to testers and smartphones. Figure 11 shows the localization results for three different users. The results reveal that the difference in testers’ heights and walking patterns has an effect on the localization performance. Figure 12 shows the localization results for two smartphones, from which we can see that a slight difference exists between the localization performances of the two smartphones.

**Figure 13:**
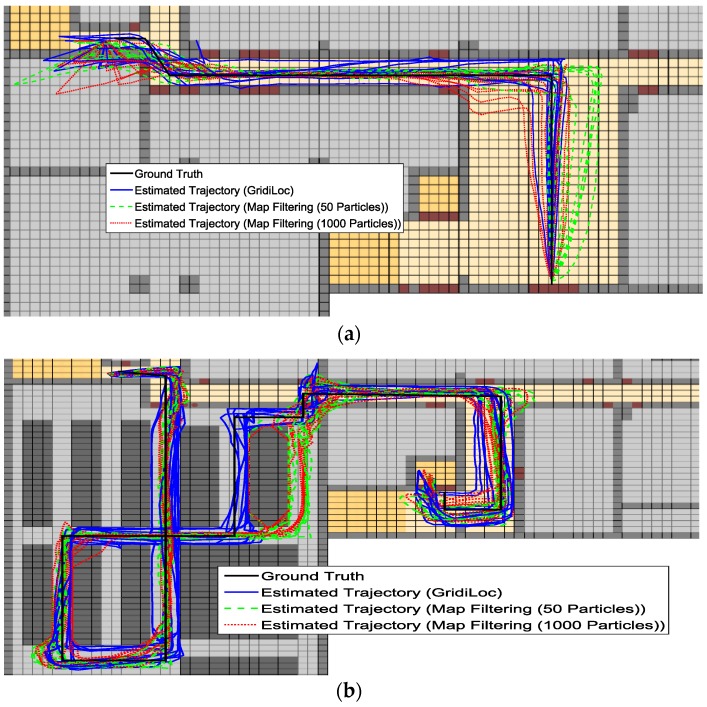

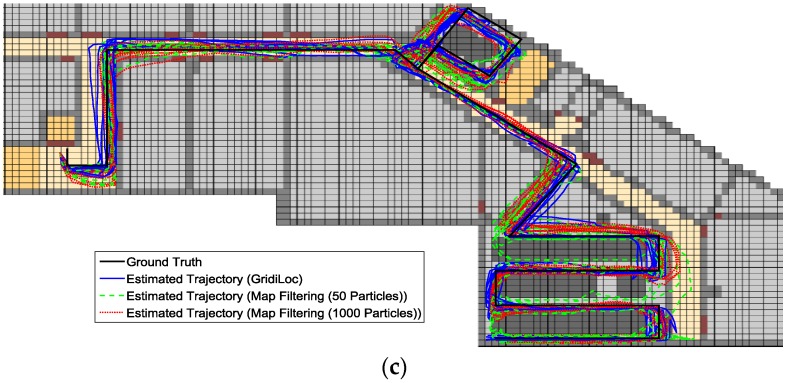
Estimated trajectories along the first test path. (**a**) Estimated trajectories on the first floor; (**b**) estimated trajectories on the second floor; (**c**) estimated trajectories on the fourth floor.

**Figure 14:**
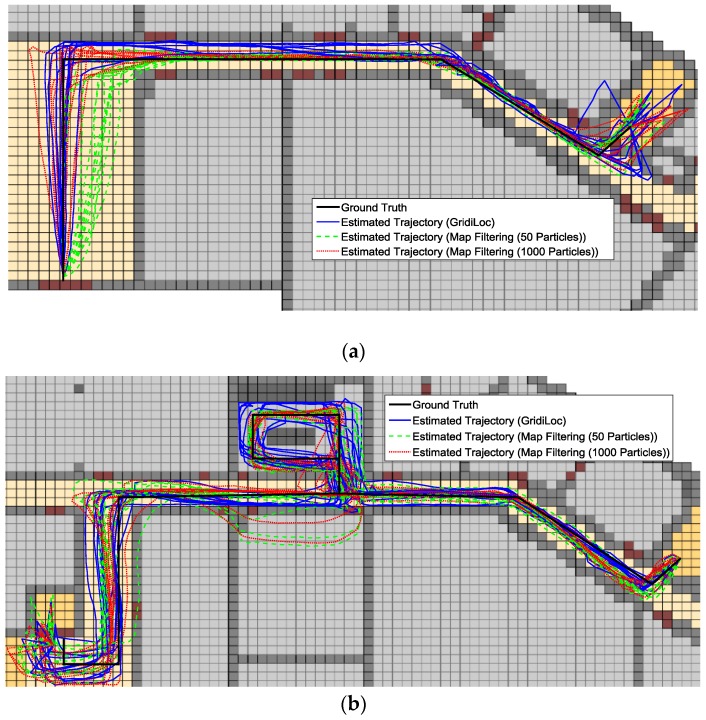

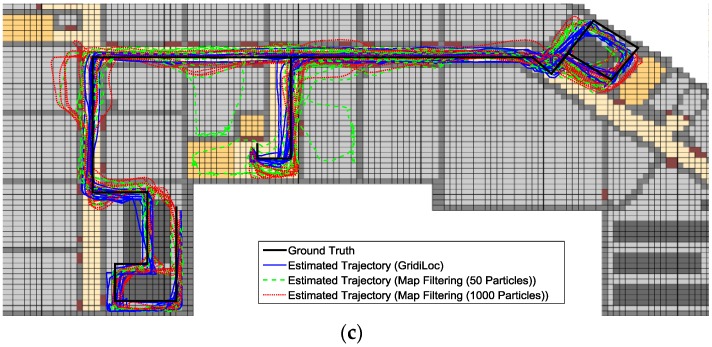
Estimated trajectories along the second test path. (**a**) Estimated trajectories on the first floor; (**b**) estimated trajectories on the second floor; (**c**) estimated trajectories on the fourth floor.

Figure 13a–c illustrates the estimated trajectories by GridiLoc and the map filtering approaches on the different floors along the first testing path. Similarly, Figure 14a–c shows the estimated trajectories along the second testing path.

From Figure 13 and Figure 14, we can see that when tracking occurs in complex indoor environments, such as the place with several close passages, particles might propagate into erroneous passages, leading to the subsequent trajectory deviating from the ground truth. For example, in the scene of Figure 13b,c and Figure 14c, multiple close passages exist in several large rooms. Thus, tracking failure occurs frequently in these rooms. Additionally, in the fork road of a corridor environment (e.g., the rightmost fork road in Figure 13a and Figure 14c), the localization error also becomes quite large due to the increased propagation freedom for particles. Overall, GridiLoc is more reliable and accurate in these test areas because these areas normally cause the dead ending issue, which is handled via backtracking. That is, the dead ending contributes to the calibration of estimations. This is also because of the utilization of fine-grained spatial contexts represented in the grid model, enabling better assistance of PDR.

### 6.3. Localization Performance

**Figure 15:**
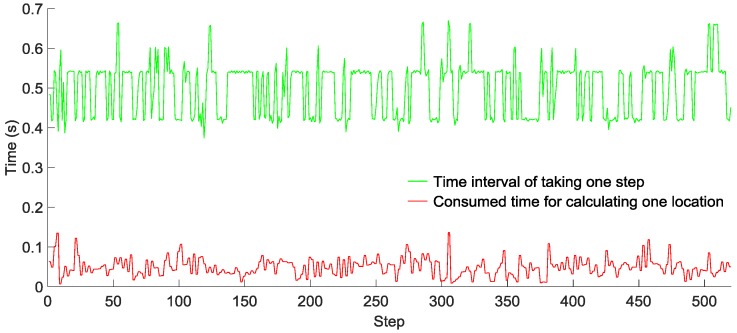
Time interval of taking one step and calculating one location for Tester 1.

**Figure 16:**
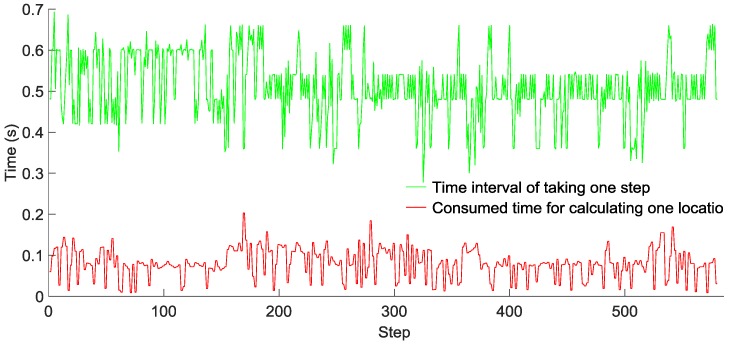
Time interval of taking one step and calculating one location for Tester 2.

To evaluate the computational complexity of our GridiLoc algorithm running on smartphones, two testers walked down a long corridor, and the time of taking one step and that of calculating one location with the grid filter were recorded, as shown in Figure 15 and Figure 16, respectively. Tester 1 walked 520 steps in 272 s, while Tester 2 walked 580 steps in 328 s. The average time of taking one step is 0.52 s for Tester 1 and 0.55 s for Tester 2, while the average time of calculating one location in two tests is 0.05 s and 0.08 s, respectively. Obviously, the time of calculating one location is much smaller than the time of taking one step. The result reveals that most of the time, the GridiLoc program is free and waiting for the incoming of sensor measurements. It also proves the low computational complexity of the proposed grid filter approach. For a backtracking process, it is *T* (*T* = 5) times long than the time of calculating one location. We believe the extra time consumption from the backtracking process is acceptable considering its great advantages in improving the localization accuracy and reliability.

### 6.4. Impact of Threshold T

In this experiment, we evaluated the impact of the threshold value *T* on localization accuracy and on the time of off-line calculating the test trajectories. *T* refers to the threshold of the time of dead ending events. In order to effectively evaluate the impact of *T*, we selected 10 tough test trajectories from the complete dataset, in which many dead ending events occurred. The 10 trajectories were re-estimated with *T* at 2, 5, 10, 13 and 15, respectively. For each value of *T*, we recorded the standard error, mean error, median error, 67% CEP (Circular Error Probable) and the average time of estimating 10 trajectories. The experimental result in Table 2 depicts that the selection of the value of *T* has a considerable impact on the localization accuracy and on the consumed time. A large value of *T* (e.g., 15) can result in the ignorance of many real dead ending events, which is a False Negative (FN). As a result, the tracking failure might occur. Furthermore, a large value of *T* increases the time consumption due to the re-estimation of *T* locations by the grid filter for each backtracking operation. Conversely, a small value of *T* (e.g., two) might cause frequent backtracking operations and misidentification of the dead ending event, which is a False Positive (FP). This can produce two main adverse consequences. One is the increase of the time consumption due to the frequent and needless backtracking operations. The other is the incorrect calibration of the estimations in the dead ending states. Accordingly, we believe a value between five and 10 is suitable to obtain a better result in terms of localization accuracy and time consumption.

### 6.5. Adaptivity of the Step Length Model

This experiment evaluated the adaptivity of the step length model. Two testers walked along a 290 m-long path 20 times in total. The parameter of the Weinberg model ranges from 0.5 to 0.7, and its performance was compared with our method. For each value of the model parameter, the inferred distance is the average value in the 20 times tests. As shown in Table 3, the best value of the model parameter varies with different testers. Specifically, the best values of the model parameter for Tester 1 and Tester 2 are 0.58 and 0.54, respectively. The Weinberg model that represents the traditional methods of calculating the step length is sensitive to the model parameter. On the contrary, the proposed method can adapt better to different people, and the initial value of the model parameter has little impact on its performance.

### 6.6. Energy Consumption

**Figure 17:**
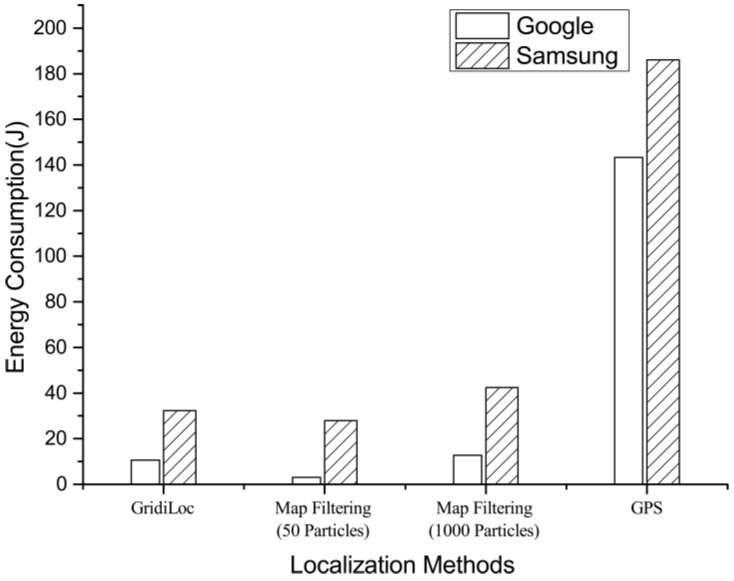
Energy consumption of different localization solutions.

This experiment evaluated the energy consumption of GridiLoc, map filtering and GPS on two android smartphones: Samsung Galaxy S Note 4 and Google Nexus 6. To test the energy consumption of GridiLoc and map filtering, two testers walked along one of the test paths illustrated in Figure 7 12 times in total. The GridiLoc app and map filtering app were deployed and ran on the two smartphones. To test the energy consumption of GPS, the two testers walked along an outdoor path 12 times in total and with the GPS program running on the two smartphones. The method proposed in [24] was utilized to record the energy consumption of a program on android smartphones. The average time of conducting one indoor or outdoor test is about 340 s. The average energy consumption of six tests for each smartphone is illustrated in Figure 17, from which we can see the map filtering approach with 50 particles consumes the least energy. Nevertheless, GridiLoc consumes less energy than the map filtering approach with 1000 particles. This is mainly because of the adaptation of the buffer mechanism with which GridiLoc just needs to calculate the probabilities of the cells in the buffer (e.g., a 7 × 7 matrix) for one estimation. On the contrary, the map filtering approach needs to calculate the probabilities of all of the particles (e.g., 1000) for one estimation. We believe the energy consumption of our method is acceptable since it achieves much higher localization accuracy than the map filtering approaches. Furthermore, our method consumes much less energy than GPS, which is widely used in navigation applications nowadays. Furthermore, many solutions can be adopted by software engineers to reduce the energy consumption of a program before bringing it to market.

## 7. Discussion

Determination of initial location: Determining users’ initial location is crucial in GridiLoc. To make the process more user-friendly, several solutions for automatic initial location determination are suggested as our future work. The first is to detect the GPS signal disappearance when a GPS-tracked user is transiting from an outdoor environment to an indoor environment. Knowing the indoor map of a building, the user’s current indoor location can be pinpointed at the entrance of the building. Unfortunately, it will fail if a user is in the indoor environment at the beginning. In addition, a particle filter-based method can also be used to infer the initial location [25]. Its drawback is that the user needs to walk a long distance before his or her current location converges to a single and small cluster of particles. The third solution leverages on Wi-Fi Access Points (APs) to determine users’ initial location, which seems more straightforward. However, the precursor is the availability of the coarse locations of Wi-Fi APs and/or fingerprints. Combining these solutions might have the problem well solved. For instance, when a user is tracked from an outdoor environment to an indoor environment, the first solution can be adopted. If a user is in an indoor environment at the beginning, with the availability of the coarse location of Wi-Fi APs, the fusion of the second and the third solution can reduce the required time for convergence.

Human behaviors: The diversity in users’ behavior may result in varying readings of smartphone sensors and thus affects the location estimation of PDR. This paper considers four kinds of common motions, including walking, staying static, climbing up or down stairs and taking an elevator. It is affirmative that humans can have various poses with at least 244 degrees of freedom [26]. Additionally, users may sometimes behave unpredictably. Therefore, it is difficult to robustly and accurately detect mobility information merely using phone sensors. Wi-Fi and other wireless techniques can be utilized to infer users’ movement information and are less affected by users’ behaviors. Thus, a combination of wireless techniques and sensors might be an optimal solution for motion determination.

Cell size: The granularity or size of grid cells has an impact on the localization accuracy and computational load. The commonly-used size of the grid filter for localization is between 0.1 m and 1 m according to the literature [12]. A size of 0.7 m is used in this paper because the distance a user normally traveled in one step is around 0.7 m. If we choose a larger cell size (e.g., 1 m), the rule that a user’s location should be always at the center of a cell will lead to inaccurate estimations. A grid model with a smaller cell size (e.g., 0.1 m) provides more fine-grained location data and can achieve better localization, but it may exponentially increase query processing time (e.g., shortest path queries), thus leading to performance and scalability problems.

## 8. Conclusions

In this paper, we propose and evaluate GridiLoc, a reliable and accurate indoor pedestrian tracking approach that fuses smartphone sensors and the fine-grained spatial contexts represented in a grid model by using a backtracking grid filter, without the need for infrastructure assistance. By using the historical tracking data and a topological graph, GridiLoc can better aid PDR and mitigate the dead ending issue. Furthermore, when the dead ending is caused by an erroneous step length model, the model parameter can be automatically calibrated. Furthermore, we introduce the buffer mechanism and a topological graph, which can dramatically reduce the computational complexity of GridiLoc. The experimental results in a multi-floor environment reveal that compared to the traditional map filtering algorithm, GridiLoc is more effective in reducing the frequency of tracking failures and achieves a better accuracy of 2.5 m 95% of the time. Additionally, extra experiments were conducted, and the results show a great improvement in model adaptivity compared to the Weinberg method, as well as an acceptable computational complexity and energy consumption of GridiLoc.

With regards to limitations and future works, there are several items we will investigate. First, the computational complexity and storage load of GridiLoc can be further reduced from two aspects. One is to calculate the shortest path distance on a server in an off-line manner. The other is to design a multi-level cache mechanism that loads and requests the grid model and the shortest path distance in a dynamic way. The second challenge that still awaits being solved is how to obtain accurate heading estimation, which is independent of different phone poses (e.g., phone in the pocket, users swing the phone when moving) and motion states. Third, we plan to describe more semantic contexts in the grid model for limiting users’ movement and improving the location estimation. For example, an open-plan office shared by several research groups can be divided into different function areas based on different research groups, and the probability of users wandering around their own group is much higher than around the other groups.

## Figures and Tables

**Table 1:** Four error measures for different algorithms. CEP, Circular Error Probable.

	GridiLoc	Map Filtering (1000 Particles)	Map Filtering (50 Particles)
Standard error	0.79 m	1.19 m	3.53 m
Mean error	1.27 m	1.68 m	2.17 m
Median error	1.12 m	1.38 m	1.39 m
67% CEP	1.45 m	1.86 m	1.94 m

**Table 2:** Impact of *T* on localization accuracy and computation time.

	T = 2	T = 5	T = 10	T = 13	T = 15
Time	113.34 s	79.21 s	88.00 s	116.84 s	122.76 s
Standard error	2.36 m	0.92 m	0.87 m	0.89 m	8.55 m
Mean error	1.87 m	1.45 m	1.34 m	1.40 m	4.57 m
Median error	1.23 m	1.22 m	1.15 m	1.19 m	1.61 m
67% CEP	1.66 m	1.64 m	1.52 m	1.56 m	2.16 m

**Table 3:** Comparison of Weinberg model and proposed method.

Step Length Model Parameter	Tester 1		Tester 2	
	Weinberg Distance (m)	GridiLoc Distance (m)	Weinberg Distance (m)	GridiLoc Distance (m)
0.5	255.3	286.6	266.2	285.1
0.54	273.7	290.4	286.4	292.5
0.58	294.5	284.8	305.5	293.1
0.62	314.1	292.1	325.7	289.5
0.66	334.5	282.9	346.3	291.3
0.7	354.9	285.8	365.6	294.8
